# Complete Genome Sequence of Mameliella alba Strain KU6B, a Cyclohexylamine-Utilizing Marine Bacterium

**DOI:** 10.1128/MRA.00273-20

**Published:** 2020-05-07

**Authors:** Taisei Yamamoto, Yaxuan Liu, Yoshie Hasegawa, Hiroaki Iwaki

**Affiliations:** a Department of Life Science & Biotechnology, Kansai University, Suita, Osaka, Japan; University of Southern California

## Abstract

Here, we report the complete genome sequence of Mameliella alba strain KU6B, a bacterium newly isolated from seawater of Boso Peninsula in Japan that is capable of utilizing cyclohexylamine. The complete genome contained a 5,386,988-bp circular chromosome and three circular plasmids of 256,516, 112,434, and 76,727 bp.

## ANNOUNCEMENT

The cyclohexylamine (CHAM) degradation pathway is potentially useful for not only bioremediation but also synthetic organic chemistry. In particular, the flavin-containing CHAM oxidase from Brevibacterium oxydans IH-35A has been shown to have synthetic potential for industrially important chiral amines (1–7). To date, CHAM-utilizing bacteria have been isolated only from terrestrial sites.

Here, we report the complete genome sequence of Mameliella alba KU6B, a marine CHAM-utilizing bacterium, in order to improve our knowledge of the biocatalytic potential of marine bacteria.

*M. alba* KU6B was isolated from surface seawater collected from Boso Peninsula in Chiba, Japan (latitude, 34.9; longitude, 134.89). Briefly, marine bacteria were collected from 1 liter of seawater by filtration and were suspended in Daigo’s IMK-SP (Nihon Pharmaceutical) as described previously (8). A 1-ml suspension of the sample was inoculated into 4 ml Daigo’s IMK-SP supplemented with 5 mM CHAM as a carbon source and incubated at 25°C on a rotary shaker at 100 rpm. After 7 days of enrichment, 4 μl of the culture medium was transferred to fresh medium and incubated for 7 days. Strain purification was performed as described previously (9). The isolate KU6B did not grow on Daigo’s IMK-SP without CHAM, indicating that KU6B utilizes CHAM as a sole source of carbon.

To isolate the genomic DNA, *M. alba* KU6B was cultured for 2 days in 50 ml marine agar 2216 (Becton, Dickinson) at 25°C. Cells were washed with Tris-EDTA buffer and then resuspended in 15 ml of the same buffer. Genomic DNA was isolated using Wilson’s procedure (10).

A 20-kb SMRTbell template library was prepared and sequenced using a PacBio RS II instrument (Pacific Biosciences), and subreads were filtered, as described previously (11). Default parameters were used for all software, unless otherwise noted. In total, 137,582 reads, composed of 1,517,719,281 bp, with an *N*_50_ value of 15,913 bp, were obtained. The reads were *de novo* assembled with the Hierarchical Genome Assembly Process (HGAP) protocol version 3 in SMRT Analysis software (12) to produce four circular contigs totaling 5,832,665 bp. The genome sequence was annotated with DFAST (https://dfast.nig.ac.jp) (13). The basic genome characteristics are shown in Table 1.

**TABLE 1:** General genomic characteristics of Mameliella alba KU6B

Genome feature	Length (bp)	G+C content (%)	Coverage (×)	No. of coding sequences	No. of rRNAs	No. of tRNAs	GenBank accession no.
Chromosome	5,386,988	65.1	187	5,531	9	60	AP022337
pKUB257	256,516	63.1	156	279	0	2	AP022338
pKUB112	112,434	62.7	313	124	0	0	AP022339
pKUB77	76,727	59.9	83	75	0	0	AP022340
Total	5,832,665	64.9	186	6,009	9	62	

Strain KU6B was identified using 16S rRNA gene sequence analysis, digital DNA-DNA hybridization (dDDH) (14, 15), and average nucleotide identity (ANI) (16), as described previously (11). The 16S rRNA gene sequence of strain KU6B was most similar to that of the type strain of *M. alba*, exhibiting 99.50% identity, and dDDH and ANI values compared with the type strain of *M. alba* were 84.4% and 97.8%, respectively. The values exceeded the proposed species boundary values (16, 17), suggesting that strain KU6B is a novel strain of the known species *M. alba*.

To identify genes related to CHAM degradation, we performed BLAST searches using *in silico* molecular cloning software (*in silico* biology) and the CHAM-, cyclohexanone-, and cyclopentanone-degrading genes of terrestrial bacteria (3, 18) as the query sequences. Using these data, we identified the gene cluster-encoding enzymes involved in the complete oxidation of cyclohexanone to adipate at nucleotide positions 59336 to 71649 of plasmid pKUB77. However, we could not determine the gene responsible for the oxidation of CHAM to cyclohexanone, indicating the presence of a novel CHAM-oxidation enzyme.

This complete genome sequence will facilitate the identification of the CHAM-oxidation enzyme and provide insights into the CHAM-degradation pathway in marine bacteria and evolutionary aspects of marine CHAM degraders.

### Data availability.

The genome sequence of Mameliella alba strain KU6B is available from DDBJ/EMBL/GenBank with accession numbers AP022337, AP022338, AP022339, and AP022340. The associated BioProject, BioSample, and Sequence Read Archive accession numbers are PRJDB9188, SAMD00200669, and DRR205090, respectively.
